# Amelioration of Single Clove Black Garlic Aqueous Extract on Dyslipidemia and Hepatitis in Chronic Carbon Tetrachloride Intoxicated Swiss Albino Mice

**DOI:** 10.1155/2018/9383950

**Published:** 2018-05-02

**Authors:** Gia-Buu Tran, Sao-Mai Dam, Nghia-Thu Tram Le

**Affiliations:** Institute of Biotechnology and Food Technology, Industrial University of Ho Chi Minh City, 12 Nguyen Van Bao Street, Ward 4, Go Vap District, Ho Chi Minh City, Vietnam

## Abstract

Single clove garlic is the product of atypical bulbing process of garlic under specific conditions. Therefore, the number of researches on single clove garlic bioactivity is limited. Recently, the hepatoprotective effect of single clove garlic has been demonstrated. In this study, we investigated amelioration of single clove black garlic aqueous extract, a processed product from single clove garlic, on dyslipidemia and hepatitis induced by chronic administration of CCl_4_. Mice were randomly divided into four groups: control, extract control, CCl_4_ intoxication, and coadministrated CCl_4_ and extract group. Mice were orally given a dose of 1 ml/kg body weight of CCl_4_ for 28 days twice a week to establish chronic liver injury model. To evaluate the hepatoprotective effect of single clove black garlic, mice were cotreated with CCl_4_ and single clove black garlic extract (200 mg/kg body weight) via gastric gauge for 30 days. Cotreatment with CCl_4_ and extract could improve the changes of body weight, liver weight, and relative liver weight as compared to CCl_4_ intoxicated mice. Single clove black garlic ameliorated dyslipidemia and the elevation of ALT and AST levels induced by chronic CCl_4_ intoxication. Histological studies revealed that single clove black garlic could prevent mononuclear cells infiltration and hepatocyte necrosis.

## 1. Introduction

Liver is the vital organ in human body due to its important role in metabolism of endogenous and exogenous molecules, such as lipids, proteins and carbohydrates, and its detoxification functions. Liver is vulnerable to a variety of liver diseases including hepatic steatosis, hepatitis, fibrosis, cirrhosis, and hepatocellular carcinoma [1, 2]. It has been suggested that free radicals, reactive oxygen species, and lipid peroxidation serve a pivotal role in pathogenesis of liver diseases [3]. Therefore, antioxidant activity is considered as the key mechanism underlying the protective effect of traditional medicines which prevent and ameliorate hepatic damage in chronic liver diseases. Therefore, a large number of researches investigating identification and isolation natural hepatoprotective compounds have been documented in recent years [4].

For screening potential hepatoprotective medicines, toxic chemicals and xenobiotics such as carbon tetrachloride, thioacetamide, paracetamol, and alcohol have been used to generate pathological models [5–8]. Among these substances, carbon tetrachloride is the most common used toxicant since it provides an ideal model for studying oxidative hepatic damage due to its distinctive hepatotoxicity and its rapid metabolisms. Furthermore, carbon tetrachloride has also been used in industry such as refrigerant, fire suppression agent, and cleaning solvent; thus the risk of CCl_4_ exposure has been considered. In liver, carbon tetrachloride is metabolized by hepatic cytochrome P450 leading to production of hepatotoxic metabolites such as trichloromethyl and peroxy radicals, which lead to lipid peroxidation, alteration of cell membrane permeability, and cell death [9]. It has been well documented that CCl_4_ administration not only leads to fatty liver and hepatocyte necrosis, but also induces accumulation of triglycerides, decrease of reduced glutathione level, membrane damage, and loss of enzyme activity [9]. Furthermore, Hsu and collaborators also suggested that carbon tetrachloride induced liver cirrhosis response was similar to human liver cirrhosis [10].

Garlic (*Allium sativum* L.) is a traditional herbal spice and well-known functional food in Vietnamese and Asian cuisines. It has been documented that garlic possesses many bioactive compounds, such as alliin, allicin, allyl-sulfides, ajoene, and 1,2-vinyldithiin, which account for many health benefits such as anticancer, antithrombotic, anti-inflammatory, antioxidant, antimicrobial, cardioprotective, and immune-modulatory activities [11]. Obioha and collaborators have indicated that garlic exerts potential hepatoprotective effect through lowering lipid peroxidation and activating antioxidant defense system [12]. Recently, garlic also is reported for its antihyperglycemic, antihyperlipidemic, and anti-inflammatory effects in type 2 diabetes mellitus associated with obesity patients [13]. However, utilization of raw garlic is strictly limited due to its peculiar flavor and its involvement in hemolytic anemia and gastrointestinal mucosa damage [14, 15].

Black garlic, the processed product which is generated by fermentation process in high temperature and high humidity, has appeared in markets for decades. Black garlic has sweet taste and eliminates unpleasant odor of raw garlic. It possesses many bioactivities including inhibition of colon and gastric cancer cells growth, antioxidant, alteration of lipid profile in diabetes, antiobesity, anti-inflammatory, and antiallergic activities [16–18]. Furthermore, black garlic extract has proven its ameliorating effect on A*β*-induced neurotoxicity and cognitive impairment [19]. Some reports also indicate that black garlic and its bioactive constituent, S-allyl-cysteine, employ hepatoprotective effects in alcohol, D-galactosamine, high fat diet, and acute carbon tetrachloride intoxicated models [20–22]. Of note, the quality and bioactive value of black garlic are diverse depended on processing method and garlic cultivars [18, 23, 24].

Due to cultivation practices and climate conditions, the bulbs of garlic sometime are not divided into cloves and generates single clove of garlic, known as single clove garlic, solo garlic, and pearl garlic. The number of researches of single clove garlic bioactivity is limited. Recently, Naji et al. (2017) suggested that single clove garlic exerted the higher hepatoprotective effect than normal garlic, which was known as “multiclove garlic” in CCl_4_ intoxicated rabbit model [25]. Furthermore, the beneficial effect of multiclove black garlic on acute carbon tetrachloride intoxicated rat was proved [21]. To our knowledge, the effects of single-clove black garlic (SBE) on dyslipidemia and liver injury in hepatic pathology model have not been studied yet. In this study, we investigated the hepatoprotective effect of single clove black garlic aqueous extract on dyslipidemia and hepatitis induced by chronic administration of carbon tetrachloride in Swiss albino mice.

## 2. Materials and Methods

### 2.1. Chemicals

All reagents were provided by Sigma-Aldrich Chemical Company (St. Louis, MO, USA) unless otherwise noted. Carbon tetrachloride was obtained from Merck (1.02223.1000, Merck, Germany) and extra virgin olive oil was purchased from Sigma (W530191, Sigma, USA).

### 2.2. Collection and Preparation of Black Garlic Extract

Single clove garlic (*Allium sativum* L.) was cultivated from Ninh Hai District, Ninh Thuan Province, Vietnam, in March 2017. The specimen was authenticated by taxonomist at Institute of Biotechnology and Food Technology, Industrial University of Ho Chi Minh City, and voucher specimen has been deposited at local institutional herbarium for further reference.

**Figure 1:**
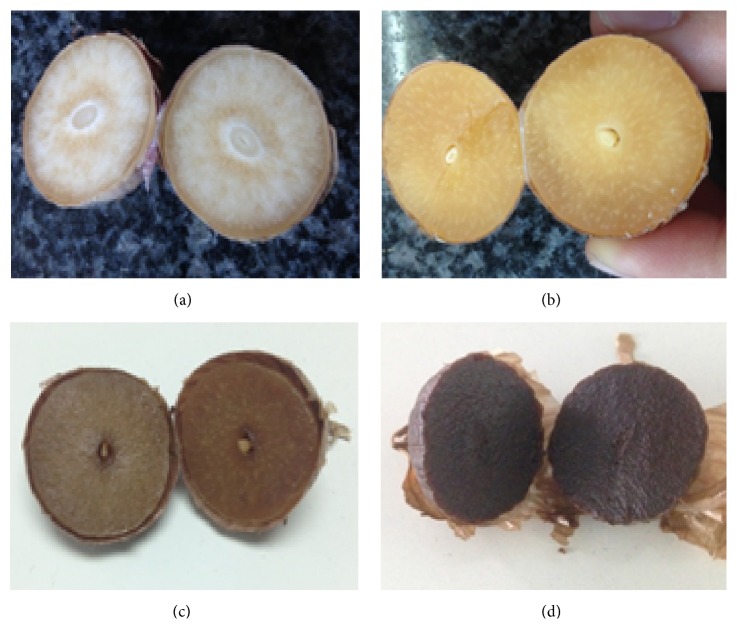
Fermentation process of single black garlic. In brief, whole bulbs of single clove garlics were fermented at 75°C in relative humidity 90% for 20 days until they obtained the particular black color. The representative samples were collected after 5 days (a), 10 days (b), 15 days (c), and 20 days (d).

The whole of single-clove garlic (diameter 15 ± 2 mm) was incubated in temperature and humidity controlled chamber (Shellab, USA) according to our institutional procedure. In brief, garlic was fermented at 75°C with 90% relative humidity for 20 days (Figure 1). The single clove black garlic extract was prepared by Shin et al. (2014) procedure with some modifications [21]. Single clove black garlic was peeled and mashed in 10 volumes of distilled water. Single clove black garlic cells were ruptured via assistant of pulsed microwave at 100 W for 5 minutes. The extract was prepared by refluxing in 105 ± 2°C for 1 hours, and then the extract was filtrated three times using Whatman number 1 filter paper. The filtrate was collected and concentrated in vacuum rotary evaporator to get 20°Bx. Subsequently, the aqueous extract was sterilized and divided into several 50 ml tubes and stored in −80°C until further use. Single clove black garlic extract (SBE) used in this study comprised 20.78 ± 0.21% solid materials, pH 3.64 ± 0.10.

### 2.3. Determination of S-Allyl-Cysteine Concentration in Aqueous Extract of Single Clove Black Garlic

For screening the presence of S-allyl cysteine, a well-known bioactive compound of black garlic in aqueous extract of single clove black garlic, we performed LC/MS analysis with the given protocol. In brief, aliquots (20 *μ*L) of the aqueous extract were subjected to HPLC Agilent 1200 infinity liquid chromatography system (Agilent Technologies, CA, USA) coupled with MicroTOF-Q mass spectrometer (Bruker Daltonics, Germany). Separation of the analytical compounds was carried out using an ACE C18 column (4.6 × 150 mm I.D., 3.5 *μ*m particle size, Advanced Chromatography Technologies, Aberdeen, Scotland, UK) at a flow rate of 0.3 mL/min. The solvent system consisted of two phases: mobile phase was composed of acetonitrile supplemented with 0.1% formic acid and water phase composed of 0.1% formic acid in water. The column oven temperature was maintained at 50°C. The mass spectrometer was operated with electrospray ionization source (ESI) at positive mode, and mass detection was performed in full scan mode in the range 50–3,000 *m*/*z*. The following parameters were applied to the instrument: capillary voltage 4,000 V, end plate offset −500 V, drying gas flow rate 10.0 l/min, the drying gas temperature 200°C, collision cell radio frequency 250.0 Vpp, and nebulizer 1.5 Bar. Data analysis was performed using Bruker Compass Data Analysis 4.0 software. The compounds were verified by comparison of the ESI-mass spectra and LC retention time of an authentic standard of S-allyl-cysteine where complete matching was observed.

### 2.4. Animal Experiment Design

Eight-week-old male Swiss albino mice were obtained from Pasteur Institute of Ho Chi Minh City, weighting approximately 30–32 g. The animals were randomly divided into polycarbonate cages with 5 mice for each cage. They were housed under standard husbandry conditions with 12 h light-dark cycle (8:00–20:00) for at least 1 week to acclimate with laboratory environment. They were supplied ad libitum with standard chow and distilled water. The experimental procedure was strictly compliance with Declaration of Helsinki (1964). Twenty healthy mice were randomly divided into 4 groups with 5 mice per group and treated as the protocol given below:

1. Group 1 (control group): mice orally received equal volume of saline for 30 days. They also received olive oil at a dose 1 ml/kg body weight via gastric gavages twice per week for 28 days.
2. Group 2 (extract control group): mice were orally received SBE (200 mg/kg body weight) for 30 days. They also received olive oil at a dose 1 ml/kg body weight via gastric gavages twice per week for 28 days.
3. Group 3 (CCl_4_ intoxicated group): hepatic injury model was induced by CCl_4_ according to previous study with some modifications [26, 27]. Mice were orally given CCl_4_ at the dose of 1 ml/kg body weight (in 50% in olive oil) twice per week via gastric gavages for 28 days. Then, they orally received equal volume of saline for last 2 days.
4. Group 4 (CCl_4_ and extract treated group): mice were treated with SBE (200 mg/kg body weight) daily via gastric gavages for 30 days. In addition, they were orally given CCl_4_ at the dose of 1 ml/kg body weight (in 50% in olive oil) twice per week via gastric gavages for 28 days. In CCl_4_ treated day, they were treated with SBE one hour before administration of mixture of CCl_4_ : olive oil.

### 2.5. Measurement of Body Weight, Liver Weight, and Relative Liver Weight

At the end of experiment (30 days), all experimental animals were fasted overnight to reduce the difference of feeding. The body weights were directly measured using an electronic balance. Subsequently, they were anesthetized using diethyl ether. Blood was collected via cardiac puncture into heparinized tube for biochemical analysis. After that, livers were collected, washed with ice-cold saline, and weighted using an electronic balance. The relative liver weights were calculated by formula (relative liver weight (%) = liver weight/body weight × 100). Subsequently, livers were immediately fixed in 10% formalin for histological studies.

### 2.6. Plasma Biochemical Analysis

Blood was collected into heparinized tubes, and then the plasma was separated by centrifuge. Lipid profile including triglycerides (TG), total cholesterol (TC), high density lipoprotein cholesterol (HDL-cholesterol), low density lipoprotein cholesterol (LDL-cholesterol), and plasma levels of hepatic enzymes such as alanine aminotransferase (ALT) and aspartate transaminase (AST) were determined using commercial diagnostic kits (Diagnosticum Zrt, Hungary) according to manufacture instructions.

### 2.7. Histological Examination of Liver

Livers were preserved in 10% formalin and processed for histological studies with Hematoxylin and Eosin staining. The specimens were dehydrated in different grades of alcohol, cleared in xylol, embedded in paraffin wax, sectioned at 4–6 um thick, and stained with Hematoxylin and Eosin. The liver sections then were examined under microscope for estimation of extent of hepatic damage.

### 2.8. Statistical Analysis

Statistical analysis was performed using Statgraphics Centurion XVI software (Statpoint Technologies Inc., Warrenton, Virginia, USA). The data were presented as mean ± standard deviation. Differences between means of different groups were analyzed using ANOVA variance analysis followed with multiple range tests, and the criterion of statistical significance was set as *p* < 0.05.

## 3. Results and Discussions

### 3.1. Determination of S-Allyl-Cysteine Content in Single Clove Black Garlic Extract

**Figure 2:**
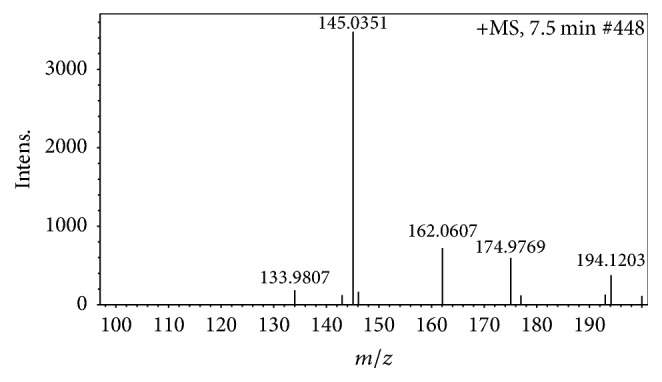
The mass spectrometry diagram of S-allyl-cysteine in single clove black garlic extract (7.5 min). The presence of bioactive compound such as S-allyl-cysteine was confirmed by LC/MS. S-Allyl-cysteine exhibited 2 peaks with *m*/*z* about 145.03 [M-H_2_O+H] and 162.06 [M+H]. Thereby, we hypothesized that SBE also employed the hepatoprotective effect in liver injury induced by carbon tetrachloride administration in mice.

There is a growing body of evidences linking black garlic with amelioration of human diseases, especially in liver disease. In previous study, Seo et al. (2009) suggested that black garlic extract could improve the lipid profile in type 2 diabetes mellitus [16]. Moreover, Jung et al. (2014) reported that consumption of black garlic extract increased HDL-cholesterol as well as ratio of LDL-cholesterol/lipoprotein B and exerted cardioprotective effect in hypercholesterolemic patients [28]. Kim et al. (2011) also proved that black garlic extract could protect liver from liver damage induced by chronic alcohol consumption [20]. Hepatoprotective effect of black garlic in carbon tetrachloride, D-galactosamine, and high fat diet treated rodents was demonstrated in previous report [21]. However, single clove black garlic aqueous extract composition and the effect of long-term administration have not been considered yet. S-Allyl-cysteine, an important sulfur-containing constituent of garlic, accumulates during manufacturing process of black garlic and exerts many biofunctions [29]. Therefore, S-allyl-cysteine content is an excellent indicator to evaluate the quality and bioactive value of black garlic. In this study, we examined S-allyl-cysteine concentration for primary evaluation of bioactive value of single clove black garlic produced by our institutional procedure. It has been found that S-allyl-cysteine existed in retention time about 7.5 min with 162.06 [M+H] and 145.03 *m*/*z* [M-H_2_O+H]. The mass spectra diagram at retention time 7.5 min is presented in Figure 2. S-Allyl-cysteine concentration in extract (228.46 ± 9.61 *μ*g/g dry weight) is comparable with concentration of S-allyl-cysteine concentration of black garlic juice (242.3 ± 6.1 *μ*g/g dry weight) which was produced from previous report and exhibited antidiabetic activity [30]. Furthermore, Kodai et al. (2015) have proven that S-allyl-cysteine, an important sulfur-containing constituent of black garlic, prevents liver fibrosis induced by carbon tetrachloride in rats [22]. Taken together, the data imply that single clove black garlic extract may have the beneficial effect for prevention of liver injury.

### 3.2. Effect of Single Clove Black Garlic Extract on Body Weight and Relative Liver Weight

In this study, we observed that there was no significant difference between body weight, liver weight, and relative liver weight of normal mice (36.26 ± 1.73 g, 1.58 ± 0.16, and 4.34 ± 0.24, resp.) and SBE administrated mice (37.24 ± 1.97, 1.68 ± 0.17, and 4.51 ± 0.29, resp., *p* > 0.05). These data implied that SBE administration did not cause the change of body weight, liver weight, and relative liver weight. On the contrary, administration of CCl_4_ caused a marked reduction of body weight (33.28 ± 2.06 g) along with a notable increase of liver weight (1.92 ± 0.22 g) as compared to normal mice (*p* < 0.05). Moreover, relative liver weight of CCl_4_ treated group was significantly increased versus control group (5.76 ± 0.45% versus 4.34 ± 0.24% resp., *p* < 0.05). These results were consistent with those reported in previous studies [26, 27, 31, 32]. Of note, SBE treatment resulted in the recovery of body weight (36.04 ± 1.31 g) and reversed the change of liver weight (1.53 ± 0.17 g) of CCl_4_ intoxicated mice (*p* < 0.05). A remarkable decrease of relative liver weights (4.23 ± 0.34%) was observed in CCl_4_ and SBE cotreated mice as compared to CCl_4_ treated mice (*p* < 0.05). Moreover, no significant difference of body weight, liver weight, and relative liver weight between control group and coadministered CCl_4_ and extract group was observed (Table 1). These results shed light on the protection effect of black garlic extract in the changes of body weight, liver weight, and relative liver weight induced by chronic CCl_4_ administration.

In previous study, Shin et al. (2014) also reported that administration with black garlic (200 mg/kg body weight) resulted in a significant decrease in liver weight as compared to acute CCl_4_ intoxicated rats [21]. The data implied that the protective effect of black garlic on liver weight was observed not only in acute CCl_4_ treatment but in chronic CCl_4_ treatment. Then the question has been raised that whether single clove black garlic aqueous extract could protect mice from the alteration of lipid profile of CCl_4_ intoxication.

### 3.3. Effect of Single Clove Black Garlic Extract on Plasma Lipid Profile

A growing body of evidences from previous reports showed that CCl_4_ intoxication caused alteration on plasma lipid profile [31–33]. In our study, CCl_4_ treatment resulted in significant increase of TG (195.10 ± 20.34 mg/dl), TC (212.29 ± 12.47 mg/dl), and LDL-cholesterol (124.18 ± 6.17 mg/dl) levels accompanied with a remarkable decrease of HDL-cholesterol (49.09 ± 6.41 mg/dl) versus control group (123.55 ± 16.63, 134.04 ± 8.58, 41.67 ± 2.97, and 67.66 ± 4.46 mg/dl, resp., *p* < 0.05). Of note, coadministration SBE and CCl_4_ to mice caused a significant decrease in plasma TG, TC, and LDL-cholesterol accompanied with a notable increase in plasma HDL-cholesterol level (*p* < 0.05) in comparison with CCl_4_ group. Furthermore, SBE could improve the LDL-cholesterol level in CCl_4_ treated mice (*p* < 0.05) but LDL-cholesterol level in coadministered CCl_4_ and SBE group was higher than control group (58.66 ± 6.58 mg/dl versus 41.67 ± 2.97 mg/dl, resp., *p* < 0.05). We also observed a significant difference in plasma total cholesterol levels between coadministered CCl_4_ and SBE mice versus normal mice (151.51 ± 11.93 mg/dl versus 134.04 ± 8.58 mg/dl, resp., *p* < 0.05). Collectively, these results suggested that black garlic extract administration could improve in lipid profile of CCl_4_ intoxicated mice and decreased plasma TG level in control mice (Table 2). Furthermore, Asdaq (2015) proved that aged garlic extract and its constituent, S-allyl-cysteine, exerted antioxidative and hypolipidemic effects on high fat diet treated rats [34]. Therefore, the high concentration of S-allyl-cysteine in extract may elucidate the improvement of single clove black garlic on the alteration of blood lipid profile in SBE and CCl_4_ treated. To our knowledge, the effect of single clove black garlic extract on lipid profile in CCl_4_ intoxication has not been elucidated yet. Thereby, these results proved in the first time the amelioration of single clove black garlic on the change of lipid profile induced by chronic CCl_4_ administration

Furthermore, there is no difference in TC, LDL-cholesterol, and HDL-cholesterol between control group and extract control group. However, chronic administration of black garlic extract reduced plasma TG level as compared to control group (89.32 ± 11.83 mg/dl and 123.55 ± 16.63 mg/dl, resp., *p* < 0.05). This finding was consolidated by the results from Ha et al. study (2015). In that report, Ha and colleagues revealed that feeding with 1.5% black garlic extract and high fat diet caused a decrease in plasma triglyceride level as compared to normal mice and high fat diet treated mice [17]. Therefore, the data implied that single clove black garlic aqueous extract has beneficial effect on lipedema not only in intoxication but also in normal physiological condition.

### 3.4. Effect of Black Garlic Extract on Plasma AST and ALT Levels

Hepatocellular toxicity is characterized by elevation of alanine transaminase (ALT) and alanine transaminase (AST), both enzymes involved in the transfer of amino groups of aspartate and alanine to ketoglutaric acid [35]. The concentration of ALT and AST in plasma of all experimental mice was presented in Table 3. In our study, levels of ALT and AST in normal mice (54.84 ± 5.86 and 146.22 ± 29.60 U/l, resp.) and SBE control mice (58.42 ± 7.13 and 141.58 ± 21.47 U/l, resp.) showed no significant difference (*p* > 0.05). These data proved that SBE administration did not cause the elevation of ALT and ALT, two markers of liver injury. On the contrary, ALT and AST levels of CCl_4_ treated group (773.88 ± 126.62 and 833.44 ± 175.56 U/l, resp.) were remarkably elevated as compared to control group (*p* < 0.05). Moreover, ALT and AST levels of coadministration of SBE and CCl_4_ mice (104.94 ± 14.48 U/l and 244.90 ± 40.86, resp., *p* < 0.05) were significantly lower than ALT and AST levels of CCl_4_ treated mice (773.88 ± 126.62 and 833.44 ± 175.56 U/l, resp., *p* < 0.05). These results indicated that single clove black garlic extract attenuated the increase of plasma ALT and AST levels induced by CCl_4_ intoxication. It has been proven that black garlic treatment ameliorated the elevation of AST and ALT induced by acute CCl_4_ injection [21]. Taken together, the data demonstrated that administration of single clove black garlic aqueous extract ameliorated hepatocyte toxicity induced by chronic CCl_4_ treatment. Furthermore, Kodai et al. (2007) reported that S-allyl-cysteine, the main component of black garlic, could invert the elevation of ALT in CCl_4_ induced acute liver injury model [36]. The lowering effects of S-allyl-cysteine and black garlic on blood ALT and AST levels were also determined in high fat diet mice [34]. As the consequence, the presence of S-allyl-cysteine in extract may account for the beneficial effect of single clove black garlic extract on ALT and AST levels in cotreated single clove black garlic extract and CCl_4_ group.

### 3.5. Histological Analysis

**Figure 3:**
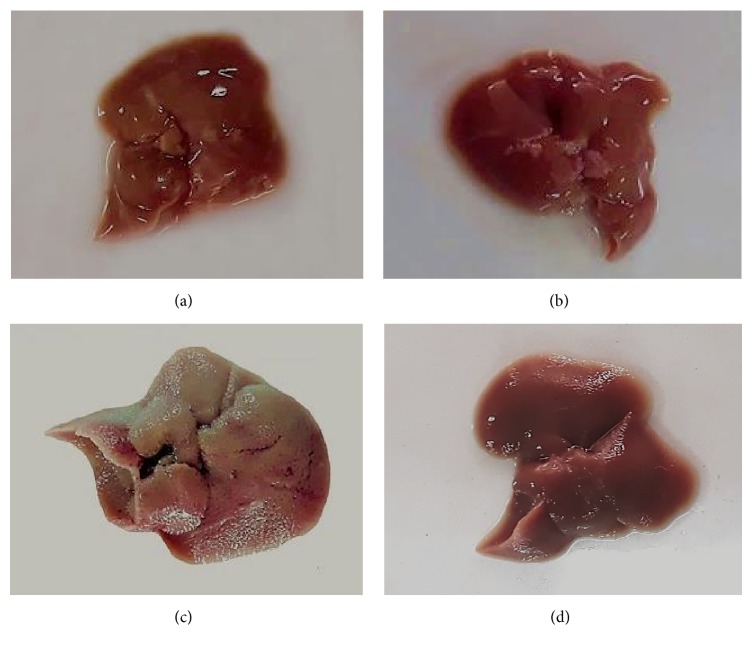
Effect of black garlic extract on gross morphology of liver from experimental mice. In gross examination, livers of normal mice showed a normal appearance with redness and soft texture, and glistering and smooth surface (a). SBE control groups showed no remarkable difference in gross morphology with livers from normal mice (b). Livers of CCl_4_ intoxicated mice revealed an enlargement of livers and changed in color (pale-brown), hardness (hard texture), and coarse surface of livers as compared with livers from control group (c). Livers of coadministered CCl_4_ and SBE mice improved the redness and hardness (semihard texture) but appeared slightly coarse surface comparing with CCl_4_ intoxicated mice (d). Our data support the strong evidence of protective effect of black garlic extract on the change of liver morphology in CCl_4_ treated mice.

The histological analysis was conducted via HE staining and gross examination for further confirmation of hepatoprotective effects of single clove black garlic extract. In gross examination, livers of normal mice showed a normal appearance with redness and soft texture, and glistering and smooth surface. There is no remarkable difference in gross morphology between livers from normal and SBE control groups. CCl_4_ intoxication resulted in an enlargement of livers and changes in color (pale-brown), hardness (hard texture), and coarse surface of livers versus the livers from control group. Livers of coadministered CCl_4_ and SBE mice improved the redness and hardness but showed slightly coarse surface comparing with CCl_4_ intoxicated mice. The representative pictures of gross examination of livers from normal, SBE control, CCl_4_ intoxicated, and coadministered CCl_4_ and SBE mice are presented in Figure 3.

**Figure 4:**
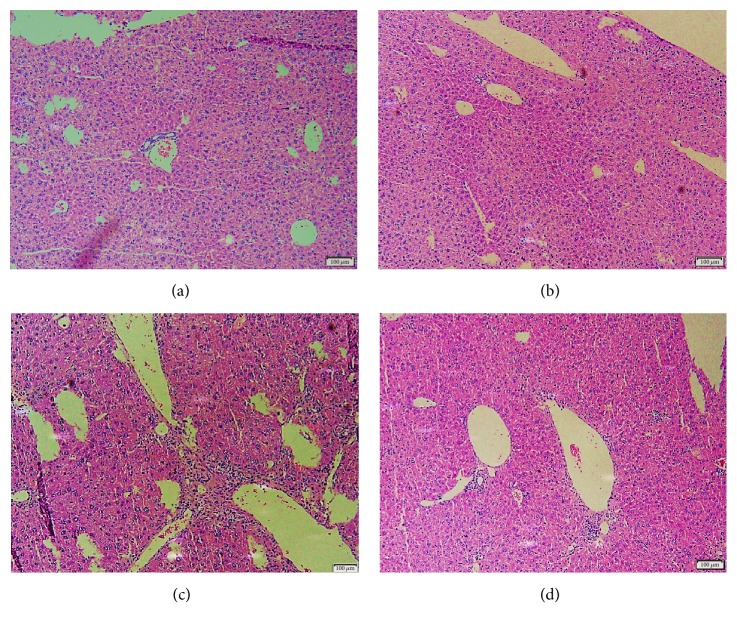
Effect of black garlic extract on liver histology from experimental mice. Liver sections from normal stained with HE revealed normal and steady architecture of hepatic parenchymal cells and portal space (a). There is no significant difference between liver sections from normal and SBE control mice stained with HE (b). CCl_4_ intoxication led to severe hepatic inflammation with mononuclear cell infiltration surrounding hepatic veins accompanied by adjacent hepatocyte necrosis (c). Of note, treatment of SBE ameliorated inflammation response with mild mononuclear cell infiltration surrounding portal space with normal architecture of hepatocytes (d). The data demonstrated that black garlic extract attenuated hepatotoxicity and liver inflammation induced by CCl_4_ administration. The scale bar is presented as 100 *μ*m.

In microscope, liver sections from normal and SBE control mice stained with HE revealed normal and steady architecture of hepatocytes and portal space. CCl_4_ intoxication led to severe hepatic inflammation with mononuclear cell infiltration surrounding hepatic veins accompanied by partial destruction of adjacent hepatocytes. On the contrary, treatment of SBE improved inflammation response with mild mononuclear cell infiltration surrounding portal space and normal architecture of hepatocytes and portal space (Figure 4). These data demonstrated that black garlic extract attenuated hepatotoxicity and hepatitis induced by CCl_4_ administration. In previous study, Kodai et al. (2007) proved that administration of S-allyl-cysteine could attenuate hepatic cell necrosis and inflammation in acute liver injury induced by CCl_4_ [36]. Furthermore, Anandasadagopan et al. (2017) reported that S-allyl-cysteine treatment could downregulate the expression of p65-NF-kB, TNF-*α*, and iNOS and suppressed liver inflammation in chromium- (VI-) induced hepatotoxicity model [37]. Therefore, high concentration of S-allyl-cysteine of single clove black garlic extract may elucidate the beneficial effect of single clove black garlic extract for liver histology in coadministrated CCl_4_ and single clove black garlic extract group.

## 4. Conclusion

In conclusion, our results demonstrated that single clove black garlic aqueous extract exerted the beneficial effects on dyslipidemia and hepatitis induced by chronic carbon tetrachloride administration, a common toxin induced liver disease model, and provided more information about the bioactivity of single clove black garlic.

## Figures and Tables

**Table 1:** Effect of SBE on the body weight, liver weight, and relative liver weight.

	Control (*n* = 5)	SBE control (*n* = 5)	CCL_4_ treated (*n* = 5)	CCl_4_ + SBE treated (*n* = 5)
Body weight (g)	36.26 ± 1.73^a^	37.24 ± 1.97^a^	33.28 ± 2.06^b^	36.04 ± 1.31^a^
Liver weight (g)	1.58 ± 0.16^a^	1.68 ± 0.17^ab^	1.92 ± 0.22^b^	1.53 ± 0.17^a^
Relative liver weight (%)	4.34 ± 0.24^a^	4.51 ± 0.29^a^	5.76 ± 0.45^b^	4.23 ± 0.34^a^

^a, b^Values with different letters within the row are significantly different (*p* < 0.05).

**Table 2:** Effect of SBE on lipid profile in experimental mice.

	Control (*n* = 5)	SBE control (*n* = 5)	CCL_4_ treated (*n* = 5)	CCl_4_ + SBE treated (*n* = 5)
Triglyceride (mg/dl)	123.55 ± 16.63^a^	89.32 ± 11.83^b^	195.10 ± 20.34^c^	121.64 ± 15.76^a^
Total cholesterol (mg/dl)	134.04 ± 8.58^a^	130.24 ± 7.32^a^	212.29 ± 12.47^b^	151.51 ± 11.93^c^
HDL-cholesterol (mg/dl)	67.66 ± 4.46^a^	70.86 ± 3.80^a^	49.09 ± 6.41^b^	68.52 ± 2.69^a^
LDL-cholesterol (mg/dl)	41.67 ± 2.97^a^	41.52 ± 3.19^a^	124.18 ± 6.17^b^	58.66 ± 6.58^c^

^a, b, c^Values with different letters within the row are significantly different (*p* < 0.05). LDL-cholesterol: low density lipoprotein cholesterol and HDL-cholesterol: high density lipoprotein cholesterol.

**Table 3:** Effect of SBE on plasma ALT and AST levels in experimental mice.

	Control (*n* = 5)	SBE control (*n* = 5)	CCL_4_ treated (*n* = 5)	CCl_4_ + SBE treated (*n* = 5)
ALT (U/l)	54.84 ± 5.86^a^	58.42 ± 7.13^a^	773.88 ± 126.62^b^	104.94 ± 14.48^c^
AST (U/l)	146.22 ± 29.60^a^	141.58 ± 21.47^a^	833.44 ± 175.56^b^	244.90 ± 40.86^c^

^a, b, c^Values with different letters within the row are significantly different (*p* < 0.05). ALT: alanine transaminase and AST: aspartate transaminase.

## Data Availability

The data sets supporting the results of this article are included within the article and its supplementary files.

## Abbreviations

SBE: Single clove black garlic extract
TG: Triglyceride
TC: Total cholesterol
HDL-cholesterol: High density lipoprotein cholesterol
LDL-cholesterol: Low density lipoprotein cholesterol
AST: Aspartate transaminase
ALT: Alanine transaminase
HE: Hematoxylin-Eosin.

## Supplementary Materials

Supplementary Materials The raw data that demonstrated the effect of single clove black garlic extract on body weight, liver weight, and relative liver weight were available in Supplementary Data S1. The amelioration effects of single clove black garlic extract on lipid profile and hepatic enzyme were proved via data set in Supplementary Data S2 and S3. Solid material content, S-allyl-cysteine concentration, and pH of single clove black garlic extract were presented in Supplementary Data S4. S1: changes of the body weight, liver weight, and relative liver weight in experimental mice. S2: changes of lipid profile in experimental mice. S3: plasma ALT and AST levels in experimental mice. S4: concentration of S-allyl-cysteine in single clove black garlic extract.
